# Molecular Analysis of *DPY19L2*, *PICK1* and *SPATA16* in Italian Unrelated Globozoospermic Men

**DOI:** 10.3390/life11070641

**Published:** 2021-06-30

**Authors:** Fabiana Faja, Francesco Pallotti, Francesco Cargnelutti, Giulia Senofonte, Tania Carlini, Andrea Lenzi, Francesco Lombardo, Donatella Paoli

**Affiliations:** Laboratory of Seminology—“Loredana Gandini” Sperm Bank, Department of Experimental Medicine, “Sapienza” University of Rome, Viale del Policlinico 155, 00161 Rome, Italy; fabianafaja@hotmail.it (F.F.); francesco.pallotti@uniroma1.it (F.P.); francesco.cargnelutti@uniroma1.it (F.C.); giulia.senofonte@uniroma1.it (G.S.); tania.carlini@uniroma1.it (T.C.); andrea.lenzi@uniroma1.it (A.L.); francesco.lombardo@uniroma1.it (F.L.)

**Keywords:** globozoospermia, acrosome, male infertility, genetics, sequencing, sperm DNA fragmentation

## Abstract

This study aims to evaluate genetic contribution and sperm DNA fragmentation (SDF) in a cohort of 18 unrelated globozoospermic Italian men (Group G). Semen samples were assessed according to the WHO 2010 Laboratory Manual and compared with 31 fertile controls. We focused our genetic analysis on the exons of the main globozoospermia-associated genes, performing qualitative PCR to assess deletion of *DPY19L2* and sequencing to detect mutations of *SPATA16* and *PICK1*. SDF was evaluated using the TUNEL assay. In Group G, 10 patients had a complete form of globozoospermia, whereas 8 patients had a partial form. Molecular analysis revealed deletion of *DPY19L2* in six of the patients, all of them with complete globozoospermia, while no mutations were found in the examined exons of *PICK1* and *SPATA16*. TUNEL analysis showed a higher SDF% in Group G. Our findings confirm *DPY19L2* defects as the most frequent genetic alteration in Italian patients contributing to globozoospermic phenotypes. Furthermore, spermatozoa with acrosomal defects could also display high levels of SDF as a possible consequence of abnormally remodeled chromatin. The possible effect on offspring of chromatin structure abnormalities and altered DNA integrity should be carefully evaluated by clinicians, especially regarding the feasibility and safety of artificial reproductive techniques, which represent the only treatment that allows these patients to conceive.

## 1. Introduction

Infertility, defined by the World Health Organization (WHO) as the failure to achieve a pregnancy after 12 months or more of regular unprotected sexual intercourse, is a major concern in public health. Current estimates indicate that up to 15% of couples of reproductive age may be affected, with an underlying complex multifactorial etiology, resulting from the interaction of both genetic and extrinsic factors [1,2].

A rare (incidence < 0.1%) genetic cause of infertility is globozoospermia. The peculiar appearance of globozoospermic ejaculates is that of a monomorphic teratozoospermia, characterized by round-headed spermatozoa lacking an acrosome [3]. The absence of an acrosome makes spermatozoa unable to interact with the zona pellucida, leading to primary infertility [4]. Even though a high incidence of fertilization failure is described [5], intracytoplasmic sperm injection (ICSI) is considered the only treatment to achieve conception. Artificial oocyte activation (AOA) with calcium ionophore may improve ICSI outcome, but fertilization rates remain low [6].

Globozoospermic spermatozoa can be round headed, completely lacking acrosomal structures and enzymes, and unable to fertilize oocytes (type 1 globozoospermia), or show a reduced acrosome often in association with other morphological abnormalities, but still capable of fertilizing (type 2 globozoospermia) [7,8]. Additionally, a complete and partial form of globozoospermia have both been described [9].

It has been reported that round-headed spermatozoa possess a lower amount of protamines (and, conversely, more histones) than normal spermatozoa [10,11] and that globozoospermic semen samples show a higher percentage of spermatozoa with immature chromatin and DNA fragmentation [12,13,14].

Most studies investigating the genetic background of globozoospermia included North-African or Middle Eastern infertile men. In contrast to Western countries, in these geographical areas globozoospermia shows a greater incidence; in particular, a possible higher rate of consanguineous marriages may increase the expression of this autosomal recessive trait [15].

Several mutations were identified and described for their strict association with globozoospermia in humans [16]. The main genes involved are the following:

- *PICK1* (protein interacting with C kinase 1) gene is located on chromosome 22q13.1. It encodes for a membrane protein paramount for protein and vesicle trafficking. Deletion of this gene leads to round-headed spermatozoa and oligozoospermia. Liu et al. [17] discovered a homozygous missense mutation (G198A) in exon 13 of the *PICK1* gene in a Chinese family. The family members affected by this homozygous missense mutation showed infertility caused by the absence of the acrosome.
- *SPATA16* (spermatogenesis associated 16) gene is located on chromosome 3q26.31. The encoded protein is involved in acrosome biogenesis during proacrosomal vesicle transport. A homozygous mutation in this spermatogenesis-specific gene was identified in a consanguineous family [18].
- *DPY19L2* (dpy-19-like 2 (C. elegans)) gene is located on chromosome 12q14.2 and it is considered the main gene involved in the etiopathogenesis of human globozoospermia [19,20,21]. It encodes for a protein involved in anchoring the acrosome to the spermatozoa nucleus [22]. Patients without *DPY19L2* have normal or subnormal sperm concentration, indicating that this gene plays a role in spermiogenesis but not in germ cell proliferation or meiosis [20,22,23]. Furthermore, a correlation exists between the severity of the phenotype and oocyte fertilization and the type of *DPY19L2* mutation [16,20,24,25].

Nonetheless, globozoospermia represents a heterogeneous disorder and determinants of the phenotype–genotype correlation remain unclear. In Italy, this severe form of teratozoospermia has been explored only in a small caseload of unrelated men [24].

Therefore, the aim of our study was to investigate the genetic contribution of the main globozoospermia-associated genes (*SPATA16*, *PICK1* and *DPY19L2*) in 18 unrelated Italian men. To further assess a putative correlation with impaired chromatin integrity, we also evaluated sperm DNA fragmentation (SDF) in acrosomeless spermatozoa in our cohort of globozoospermic patients and compared it to fertile controls.

## 2. Materials and Methods

### 2.1. Patients

We selected 18 consecutive semen samples from 18 unrelated Caucasian men of Italian origin affected by globozoospermia (Group G) and, as controls, 31 normozoospermic Caucasian men (Group N), attending the Laboratory of Seminology - “Loredana Gandini” Sperm Bank, Department of Experimental Medicine at “Sapienza” University of Rome, for semen analysis as part of an andrological work-up for preconceptional screening.

### 2.2. Semen Analysis

Semen samples were collected by masturbation after 3–5 days of abstinence. All samples were allowed to liquefy at 37 °C for 60 min and were then assessed according to the World Health Organization (WHO) Laboratory Manual [26].

The following variables were taken into consideration: ejaculate volume (mL), sperm concentration (106 per mL), total sperm number (106 per ejaculate), progressive motility (%), and morphology (% abnormal forms).

### 2.3. DNA Extraction

Total DNA was extracted from sperm using the MasterPureTM DNA Purification Kit (Epicentre^®^, Madison, WI, USA) according to the manufacturer’s instructions. Extracted DNA was quantified by NanoDrop ND-2000 (Thermo Fisher Scientific, Waltham, MA, USA) and underwent molecular analysis.

### 2.4. PCR and Sequencing

We focused our analysis on *DPY19L2* deletion and sequencing of *SPATA16* and *PICK1*, assessing putative genetic variants of the mainly altered exons as reported in literature (see Discussion Section)). Extracted DNA was amplified with specific primers (Supplementary Table S1).

Genetic analysis was performed as follows:

- *DPY19L2*: we studied exons 1, 10, 11, 12, 20 and 22. Analysis was carried out with qualitative PCR followed by electrophoresis on 2% agarose gel.
- *SPATA16*: we analyzed exon 4. To detect any mutations, amplified samples were purified and underwent automated sequencing based on the Sanger method by using 3500 Genetic Analyser (Applied Biosystem, Waltham, MA, USA). Purification of samples was carried on with **^®^**PureLink PCR purification kit (Invitrogen, Life Tecnologies, Waltham, MA, USA). Amplification reaction occurred in a volume of 13 µL containing 1 µL of **^®^**Big Dye (Applied Biosystems, Waltham, MA, USA), 2 µL of buffer **^®^**Big Dye Terminator (Applied Biosystems, Waltham, MA, USA), 1 µL of each primer, 18 ng of DNA and the amount of DNAse free water (Ambion**^®^**, Waltham, MA, USA) needed to reach the final reaction volume. Raw data from the capillary electrophoresis were analyzed by Sequencing Analysis (Applied Biosystems, Waltham, MA, USA). Subsequently, multiple alignment of the sequences versus the template was carried out to identify point mutations using the software Geneious Prime 2020.2 (Biomatters, Ltd. L2, Auckland, New Zealand).
- *PICK1*: we investigated exon 13 by sequencing as described above.

### 2.5. Sperm DNA Fragmentation

Sperm DNA fragmentation (SDF) was evaluated using the TdT-mediated dUDP nick-end labelling (TUNEL) assay (In situCell DeathDetection Kit, Fluorescein; Roche, Basel, Switzerland) [27,28].

**Figure 1:**
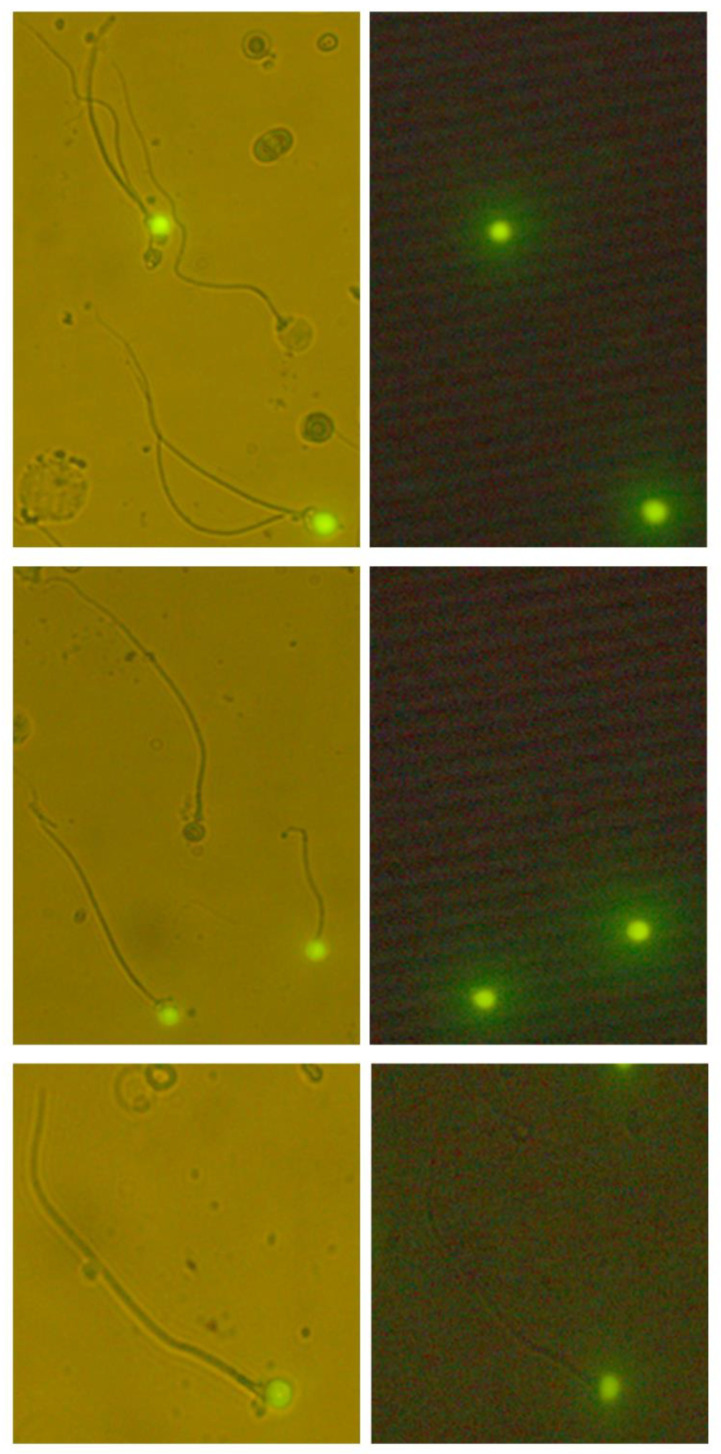
Fluorescent heads of fragmented acrosomeless spermatozoa, evaluated simultaneously using both transmitted light (bright fields) and reflected light (dark fields) (500×).

After cytological and morphological examination of sperm parameters, the samples were washed twice in phosphate-buffered saline and then cytocentrifuged (Cytospin 3; Shandon Inc., Pittsburgh, PA, USA). Samples were then analyzed using a fluorescence microscope (Leica DMR; Leica, Wetzlar, Germany), counting at least 500 cells (Figure 1).

### 2.6. Statistical Analysis

Continuous variables are presented as mean ± SD and the normality of distributions was checked using the Kolmogorov–Smirnov test; the student’s t or Mann–Whitney U test was used to compare sperm parameters between the two groups. Categorical variables are presented as counts and percentages, and comparisons were performed by Fisher’s exact test. The presence of statistically significant correlations among the considered variables was evaluated using Spearman’s rank correlation test. The probability values are 2-sided; a *p* value less than 0.05 was considered statistically significant. All computations were carried out with the Statistical Package for the Social Sciences (SPSS) 25.0 (SPSS Inc., Chicago, IL, USA).

## 3. Results

### 3.1. Semen Analysis

Comparison of the sperm parameters of Group G (Supplementary Table S2) versus Group N (age 37.2 ± 7.2 years vs. 31.8 ± 3.9 years, respectively, *p* = 0.001) revealed a significantly lower total sperm number, progressive motility and higher percentage of abnormal forms in Group G (Table 1). Moreover, in Group G morphological analysis identified 10 patients displaying complete globozoospermia with 100% round-headed acrosomeless spermatozoa and eight patients displaying partial globozoospermia with 80–95% round-headed acrosomeless spermatozoa.

With the obvious exclusion of a higher percentage of abnormal forms, the comparison of sperm parameters between men with complete and partial form of globozoospermia showed no significant differences (Supplementary Table S3).

### 3.2. Genetic Analysis

Group G molecular analysis, carried out by qualitative PCR, revealed a deletion in the *DPY19L2* gene in six out of eighteen patients (33.3%). In particular, one patient showed deletion in exon 11, one patient in exon 22 and four patients in exons 10, 12 and 22.

All patients carrying a deletion in *DPY19L2* displayed complete globozoospermia (Supplementary Table S4). In contrast, sequencing of *PICK1* and *SPATA16* revealed no mutations in the exons examined.

### 3.3. Sperm Chromatin Integrity

**Figure 2:**
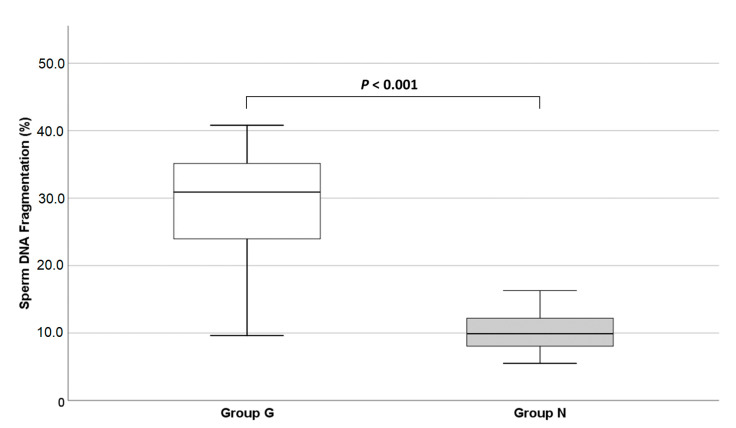
Distribution of % SDF values in the two study groups (Group G—white box, Group N—grey box) (Mann–Whitney U test, *p* < 0.001). Group G, globozoospermic men; Group N, normozoospermic controls.

A statistically significant difference in SDF was found between Groups G and N (29.7 ± 8.2% vs. 10.0 ± 2.8%, respectively, *p* < 0.001), as shown in Figure 2. Additionally, within Group G no difference in SDF was found between men with a complete or partial form of globozoospermia and between men with and without *DPY19L2* deletion.

## 4. Discussion

Globozoospermia is a severe form of teratozoospermia characterized by round-headed sperm that lack an acrosome. Sperm cells also show multiple defects, such as round nuclei, absence of the post-acrosomal sheath, separation of the nuclear membranes and, frequently, coiled tails. Other maturation defects, such as the persisting residual cytoplasmic body/droplet surrounding the nucleus or the midpiece, are often reported [4]. These morphological defects originate during spermiogenesis, specifically when the elongating head of the differentiating sperm cell should form the acrosome from the Golgi apparatus. Acrosome formation does not occur in globozoospermic patients and spermatozoa show the characteristic round-headed shape [29,30,31].

### 4.1. Sperm Parameters

In general, globozoospermia has been associated with decreased semen quality [32]. Several papers highlight the presence of a normal semen volume and concentration despite impaired total motility [4,12,33], while others indicate that both concentration and progressive motility are reduced in comparison to normozoospermic controls [9,34,35,36,37,38].

Congruent with previous studies, our data remark that globozoospermic samples show a significantly worse sperm quality compared to normozoospermic controls. Even so, the average volume, sperm concentration, total sperm number and progressive motility still fall within the 5th percentile of the WHO reference values [26].

This evidence strengthens the concept that the natural fertility failure affecting globozoospermic patients is mainly due to sperm acrosomal structural abnormalities, independent from concomitant sperm parameter alterations.

### 4.2. Genetic Analysis

The presence of consanguineous marriages in families affected with globozoospermia suggests a genetic autosomal recessive contribution to globozoospermia in humans [15,17,18,39,40,41,42,43]. However, no clear phenotype–genotype correlation has been established.

In our study we considered only the main altered exons reported in literature of the three globozoospermia-associated genes (*SPATA16*, *PICK1* and *DPY19L2*) and evaluated putative variants of these genes in a cohort of 18 unrelated men, including 10 patients affected by complete globozoospermia and eight patients affected by partial globozoospermia.

To the best of our knowledge, the present study is one of two investigating the role of genes and sperm chromatin integrity in Italian unrelated globozoospermic patients. Only Chianese et al. performed genetic screening and sperm chromatin evaluation in seven Italian globozoospermic patients [24].

Furthermore, few studies in the literature investigated genetic variants of the aforementioned genes (Table 2). While we detected no mutations of *SPATA16*, a previous paper revealed a homozygous mutation in exon 4 of *SPATA16* in three globozoospermic brothers of a consanguineous Ashkenazi Jewish family [18]. While this evidence points towards a *SPATA16* protein role in acrosome formation [44], mutant mice with the corresponding aminoacidic substitution in *Spata16* did not appear to be associated with globozoospermia, as reported in a recent study [45]. Deletion of exon 4 resulted in infertile male mice due to spermiogenic arrest, but not globozoospermia. As mice carrying the deletion experienced normal acrosome biogenesis, the authors assumed that mouse *Spata16* is not related to globozoospermia [45]. Since *SPATA16* is highly conserved in mammals, these findings could explain why no alterations of this gene were found in the globozoospermic patients examined in our study. However, analysis of *SPATA16* exon 2 revealed a new deletion in two unrelated Tunisian men, which could explain the globozoospermic phenotype [46,47].

Regarding the other globozoospermia-associated gene, *PICK1* seems to be crucial for acrosome biogenesis and a homozygous missense mutation in exon 13 of this gene resulted in round-headed acrosomeless spermatozoa in one member of a Chinese family [17].

Contrary to this evidence, our data revealed mutations neither for exon 4 of *SPATA16* nor for exon 13 of *PICK1*. However, these findings overlap with those described in Modarres et al. [48]. It should be stressed that the lack of genetic variants of *SPATA16* and *PICK1* we observed could be due to the small caseload caused by the rarity of this monomorphic teratozoospermia. Moreover, the incidence of putative genetic variants could depend on ethnicity and geographical origin examined. In addition, contributions of other uninvestigated genes on globozoospermic phenotype should not be excluded.

Among the globozoospermia-associated genes analyzed in the literature, *DPY19L2* is the most frequently mutated gene in affected patients from different ethnic and geographic origins. The most common *DPY19L2* mutation is the 200 kb deletion arising from a non-allelic homologous recombination (NAHR) between two highly similar 28 kb low copy repeats (LCRs) flanking the gene [19,20]. To support this observation, several studies have identified a recurrent homozygous deletion of the *DPY19L2* gene in complete globozoospermia, thus indicating that this gene is necessary in men for sperm head elongation and acrosome formation [19,20,21,49].

In the present study, we evaluated *DPY19L2* deletion by detecting exons 1, 10, 11, 12, 20 and 22. Analyses carried out with qualitative PCR showed that six out of eighteen patients (33.3%) displayed deletions in *DPY19L2*, including one in exon 11, another one in exon 22 and the remaining four in exons 10, 12 and 22. All six patients carrying deletions in the *DPY19L2* gene showed complete globozoospermia. Our findings agree with those in previous literature, suggesting that *DPY19L2* defects could contribute to this severe form of teratozoospermia [19,20,24,48,49,50,51,52,53,54].

Analyzing the reported percentage of *DPY19L2* mutations, together with those of the two other globozoospermia-associated genes *SPATA16* and *PICK1*, more than half of the cases carried *DPY19L2* mutations, with a rather lower frequency of *SPATA16* or *PICK1* mutations. These data highlight the role of *DPY19L2* mutations as a major cause of globozoospermia. Moreover, by comparing reported genetic data from globozoospermic patients from Western countries with reported genetic data from those from China, the frequency of *DPY19L2* variants in Chinese patients seems to be higher than that in patients from Western countries.

**Table 2:** Genetic analyses of globozoospermic patients reported in literature.

Gene	Exons Examined	Method to Identify Mutations	N° Globozoospermic Patients	N Patients Carrying Mutation	Reference
***PICK1***	exon 13	PCR and Sequencing	3 members of a Chinese family	1/3 homozygous mutated (c.1567G>A)	[17]
	all exons	PCR and Sequencing	1 Macedonian man	0/1	[50]
	exon 13	qPCR	27 Iranian men (of which 17 unrelated)	0/27	[48]
	all exons	PCR and Sequencing	4 unrelated Tunisian men (with no *DPY19L2* mutations)	0/4	[53]
	exon 13	Sequencing	18 unrelated Italian men	0/18	Present study
***SPATA16***	exon 4	Genomewide scan analysis using a 10K SNP array	3 brothers of a consanguineous Ashkenazi Jewish family	3/3 homozygous mutated (c.848G>A)	[18]
	all exons	PCR and Sequencing	1 Macedonian man	1/1 two polymorphisms (rs115897458 and rs508508)	[50]
	exon 2	PCR and Sequencing	19 (*DPY19L2* undeleted) unrelated men originating from France, Italy, Tunisia, Turkey, Libya and Morocco	2/19 (unrelated Tunisian men) deleted	[46]
	exon 4	qPCR	27 Iranian men (of which 17 unrelated)	0/27	[48]
	all exons	PCR and Sequencing	4 unrelated Tunisian men (with no *DPY19L2* mutations)	0/4	[53]
	exon 2	PCR and Sequencing	2 unrelated Tunisian men	2/2 deleted	[47]
	exon 4	Sequencing	18 unrelated Italian men	0/18	Present study
***DPY19L2***	all exons	Whole genome SNP scan	20 men (15 from Tunisia, 1 from Algeria, 2 from Morocco, 1 from Turkey and 1 from Slovenia), most of them first cousins	15/20 homozygous deleted	[19]
	exons 2, 7, 9, 10, 13, 17, 21	Genome-wide scan analysis using 10K SNP arrays	28 men (4 brothers from a Jordanian consanguineous family, 11 from France, 2 brothers from Algeria, 1 from Iran, 4 from Tunisia, 1 from Lybia, 1 from Italy, 1 from Morocco and 3 of undetermined origin)	4 Jordanian brothers: homozygous deleted for all the exons examined 4 unrelated subjects deleted	[20]
	all exons	Multiplex Ligation-dependent Probe Amplification (MLPA) and Sequencing	34 men from France and Tunisia (including 20 men described in Harbuz et al. 2011)	23/34 (67.6%) homozygous deleted 2/34 (5.9%) heterozygous deleted 9/34 (26.4%) non-deleted Point mutations identified: - exon 8: heterozygous missense mutation (c.869G>A) - exon 9: heterozygous nonsense mutation (c.1024C>T) - exon 10: homozygous missense mutation (c.1073T>A)	[21]
	exons 4, 5, 6, 7, 8, 9, 10, 11, 15, 16, 21	PCR	54 genetically independent men for all types of mutations (from 13 different countries including Iran, France, Algeria, Turkey, Morocco, Belgium, USA, Italy)	36/54 mutated (69.4%: homozygous deleted; 19.4%: heterozygous composite; 11.1%: homozygous point mutated) Point mutations identified: - exon 8: missense mutation (c.869G>A), non-synonymous mutation (c.892C>T) - exon 9: premature stop codon (c.1033C>T) - exon 15: non-synonymous mutation (c.1478C>G) - exon 21: premature stop codon (c.2038A>T) - exon 11: premature stop codon (c.1183delT)	[49]
	exons 1, 11, 22	PCR and Sequencing	2 Macedonian men	2/2 homozygous deleted	[50]
	all exons	PCR and Sequencing	15 unrelated Chinese men	4/15 homozygous deleted	[51]
	all exons	qPCR	9 men (7 from Italy and 2 from Spain)	3/9 deleted (2 homozygous, 1 heterozygous) 1/9 wild-type 5/9 point mutated (4 missense, 3 intronic and 2 synonymous)	[24]
	exons 1, 17, 22	PCR and Sequencing	5 men from Algeria (of which 3 brothers)	5/5 homozygous deleted	[52]
	exons 1, 5, 6, 7, 11, 22	qPCR	27 Iranian men (of which 17 unrelated)	20/27 deleted	[48]
	all exons	PCR and Sequencing	18 unrelated Tunisian men	11/18: homozygous deleted in exon 10 2/18: homozygous for non-synonymous mutation (c.892C>T) in exon 8 1/18: homozygous for a new splice-site mutation at the junction exon–intron 16	[53]
	exon 10	PCR and Sequencing	2 unrelated Tunisian men	0/2	[47]
	all exons	Whole-exome sequencing	9 unrelated Chinese men	5/9 deleted 4/9 with novel point mutations	[54]
	exons 1, 10, 11, 12, 20, 22	PCR	18 unrelated Italian men	6/18 deleted (1/18 in exon 11; 1/18 in exon 22; 4/18 in exons 10, 12 and 22)	Present study

### 4.3. Sperm Chromatin Integrity

To further investigate the chromatin integrity of the acrosomeless spermatozoa, we performed the TUNEL assay on semen samples of our caseload. In Group G the SDF was significantly higher than in the control group, suggesting that spermatozoa with acrosomal defects could also exhibit high levels of sperm DNA damage. On the other hand, no differences were found between complete and partial globozoospermic samples and between deleted and not deleted globozoospermic samples.

A significantly higher level of SDF in globozoospermic patients was also reported in previous literature (Table 3). However, to date only a few globozoospermic patients have been examined. Most studies used the TUNEL assay to evaluate sperm DNA integrity and their results are generally in agreement with our findings, suggesting a high alteration of sperm DNA in globozoospermia [12,24,34,35,36,37,47,55,56,57,58,59,60,61,62,63]. Only a few studies claimed that globozoospermic men had similar chromatin and DNA integrity as fertile men [33,64].

DNA fragmentation could be a consequence of abnormal chromatin remodeling, which characterizes globozoospermic samples. To support this hypothesis, some authors described abnormal chromatin condensation in globozoospermia, with a high heterogeneity in the degree of maturity [4] due to altered replacement of histones by protamines [10,40]. Protamines are necessary for proper packaging of DNA and protect sperm from DNA damage. Moreover, polymorphisms of *PRM1* and *PRM2* genes could result in reduced protamine expression associated with abnormal sperm morphology [65]. Sperm chromatin remodeling occurs simultaneously with acrosome formation during late spermatogenesis. Thus, when a protamine deficiency is present, sperm DNA damage can arise, and this could affect acrosome biogenesis which occurs in parallel to chromatin condensation. This would explain concomitant presence of an abnormal chromatin remodeling with defective acrosome in globozoospermic samples, as reported in literature.

The putative correlation between DNA damage and acrosomal deficiencies should be evaluated when patients affected by this severe form of teratozoospermia are enrolled in ICSI programs, which represent the only treatment for this type of male infertility [5]. The high level of sperm DNA fragmentation might contribute to low fertilization rate and poor pregnancy prognosis, as described by some reports [6,66,67,68]. Moreover, the possible effects of the abnormalities in chromatin structure and DNA integrity on offspring should be considered. Whenever possible, normally shaped spermatozoa should be used in partial globozoospermia.

### 4.4. Clinical Implications for Assisted Reproduction

The spread of assisted reproductive technologies since the last decade of the twentieth century allowed many globozoospermic subjects to access ICSI. The first reports indicated a greater incidence of fertilization failures compared to the “general population” [69]. Round-headed acrosomeless spermatozoa, unable to naturally interact and penetrate the zona pellucida, also appeared unable to induce oocyte activation after ICSI. In fact, plenty of studies have presented artificial reproduction attempts with globozoospermic semen samples through standard ICSI cycles, intracytoplasmic morphologically selected spermatozoa injection (IMSI), either with or without assisted oocyte activation (AOA) [70]. Fertilization and embryo development appears improved after oocyte activation, while standard ICSI without AOA may still be utilized in cases of partial globozoospermia, where a percentage of morphologically normal spermatozoa might still be present [5].

Oocyte activation is a complex network of intracellular interactions induced by oscillations in cytoplasmic calcium concentration that round-headed acrosomeless spermatozoa appear to be unable to trigger after ICSI. Therefore, AOA may potentially solve globozoospermia-induced inability to fertilize. Recreating the intracellular calcium rise, ooplasm/nuclear reactions of fertilized oocytes are artificially triggered and, thus, embryogenesis may proceed [54,70]. Despite the fact that AOA can be accomplished through different methods, both fertilization and pregnancy rates appear better than those achieved with ICSI alone [71]. Pregnancy outcomes were evaluated in a recent metanalysis by Murugesu et al., according to whom the treatment with a calcium ionophore can not only improve pregnancy and live-birth rates, but may exert positive effects on other parameters like cleavage, fertilization, blastulation and implantation [72]. Thus, this evidence has resulted in encouragement for couples and those with conditions such as globozoospemia, where success rates of ICSI alone are expected to be insufficient. In relation to globozoospermia, a number of case reports and observational studies have presented successful childbirth in cases of both total and partial globozoospermia with various forms of AOA [64,73,74,75,76,77]. However, a recent study compared fertilization and chromosomal integrity between round-headed sperm and donor sperm in oocytes from the same patient [78]. Although AOA was shown to be an effective tool for globozoospermic semen samples, the fertilization rate was lower than in donor sperm injections without AOA. On the other hand, the aneuploidy rate detected after ICSI with AOA in globozoospermic spermatozoa was comparable to ICSI with donor spermatozoa without AOA, suggesting chromosome integrity may not be affected by calcium ionophore treatment [78]. It should be emphasized that use of ICSI could allow the inheritance of mutations associated with globozoospermia, and in selected cases (for example, consanguineous marriages for recessive genes like *DPY19L2*) it might be important to seek genetic counselling to prevent this form of infertility in future progeny [79].

In conclusion, available reports are highly heterogeneous, and a thorough efficacy/safety assessment is difficult to perform and, therefore, this procedure still cannot be routinely considered [70].

## 5. Conclusions

Globozoospermia is a rare genetic cause of infertility whose phenotype–genotype correlation still remains unclear. Among the genes mainly involved, *DPY19L2* appears to play a pivotal role in contributing to globozoospermic phenotypes in patients from different ethnic and geographical origins. Furthermore, globozoospermic ejaculates seem to be characterized by a high level of sperm DNA fragmentation as a putative consequence of abnormal chromatin remodeling with an aberrant histones/protamines ratio [4,10,40].

Although ICSI represents the only treatment that allows these patients to conceive [5], the fertilization rate still remains low [6], but new treatment techniques, such as AOA, may improve both fertilization and pregnancy rates [54,70]. However, while advances in reproductive techniques have allowed these patients to conceive, the possible impacts on offspring of abnormal chromatin structure and DNA integrity should be carefully evaluated by clinicians, especially regarding the advisability and safety of using ICSI as a treatment for this form of male infertility.

## Figures and Tables

**Table 1:** Mean ± SD, median (in brackets) and significance of the sperm parameters in the two study groups (Mann–Whitney U test). Significant *p* values are in bold. Group G, globozoospermic men; Group N, normozoospermic controls.

	Semen Volume (ml)	Sperm Concentration (10^6^/^mL^)	Total Sperm Number (10^6^/ejaculate)	Progressive Motility (%)	Abnormal Forms (%)
**Group G**	2.5 ± 1.3 (2.5)	75.1 ± 69.7 (59.0)	162.6 ± 128.3 (124.5)	36.4 ± 10.8 (40.0)	94.6 ± 7.1 (100.0)
**Group N**	3.3 ± 1.2 (3.0)	84.6 ± 51.9 (75.0)	273.1 ± 188.2 (244.0)	51.5 ± 6.4 (55.0)	88.0 ± 2.9 (87.0)
***p* value**	0.07	0.254	**0.018**	**<0.001**	**<0.001**

**Table 3:** SDF rates in globozoospermic patients reported in literature. NA, Not Available; TEM, Transmission Electron Microscopy; SCSA, Sperm Chromatin Structure Assay; TUNEL, TdT-mediated dUDP Nick-End Labelling assay; SCD, Sperm Chromatin Dispersion test; AO, Acridine Orange Table A3. Chromomycin A3 staining; AB, Aniline Blue staining.

Reference	N Patients (Case vs. Control)	Method to Evaluate SDF	DNA Fragmentation Index	
			Case	Control
[55]	NA	Hoechst 33258 DNA staining TUNEL Electron Microscopy	10%	0.1%
[33]	1 vs. 2	TEM SCSA COMET	TEM: no elevated levels of apoptotic bodies SCSA and COMET: 13%	NA
[12]	1 vs. 4	TUNEL	37.0 ± 1.7%	22.5 ± 1.2%
[56]	1 (case report)	SCD	45.7% (threshold value: 30%)	/
[57]	1 vs. 1	AO	97.1%	41.3%
[58]	1 vs. 1	TUNEL	80%	27 ± 13%
[59]	2 vs. 20	TUNEL	Patient 1: 40% Patient 2: 80%	12 ± 2.12%
[60]	1 vs. unspecified	TUNEL	9.6%	1.20 ± 0.95%
[64]	1 (case report)	TUNEL	6%	/
[61]	6 vs. unspecified	TUNEL	15.5 ± 9,09%	<13%
[14]	1 (case report)	SCSA TB CMA3 AB	SCSA: 35.3% (threshold value 30%) TB: 36.6% (threshold value 35%) CMA3: 47.7% AB: 56.3%	/
[24]	8 vs. 90	TUNEL	46.92 ± 4.20%	34.04 ± 1.53%
[34]	15 vs. 21	TUNEL CMA3	TUNEL: 17.60 ± 10.72% CMA3: 65.93 ± 11.77%	TUNEL: 5.95 ± 3.02% CMA3: 21.24 ± 7.37%
[35]	20 vs. 40	SCSA CMA3	SCSA: 33.83 ± 3.64% CMA3: 49.70 ± 4.88%	SCSA: 16.31 ± 1.58% CMA3: 30.97 ± 1.71%
[36]	30 vs. 22	TUNEL	19.21 ± 3.75%	8.58 ± 1.12%
[37]	27 vs. 30	SCD TUNEL AB TB CMA3 AO	SCD: partial globo 61.7 ± 13.2; total globo 57.8 ± 11.4 TUNEL: partial globo 12.3 ± 9.2; total globo 18.3 ± 10.1 AB: partial globo 81.3 ± 10.6; total globo 79.8 ± 12.9 TB: partial globo 73.1 ± 16; total globo 86.3 ± 9.1 CMA3: partial globo 60.1 ± 13.9; total globo 68.6 ± 11 AO: partial globo 33.2 ± 26.4; total globo 30.1 ± 18.4	SCD: 11.5 ± 6.2 TUNEL: 5.9 ± 3 AB: 24.2 ± 16.2 TB: 32.8 ± 18.6 CMA3: 26.1 ± 11.6 AO: 11.5 ± 7.5
[47]	8 vs. 25	TUNEL	41.58 ± 10.99%	10.25 ± 3.83%
[62]	10 vs. 30	TUNEL	23.6 ± 5.67%	5.24 ± 1.23%
[63]	1 vs. 3	AB AO TEM	AB: 78 ± 2.65% AO: 22.33 ± 2.52% TEM: chromatin compromised	NA
Present study	18 vs. 31	TUNEL	29.7 ± 8.2%	10.0 ± 2.8%

## Data Availability

The datasets analyzed during the current study are available from the corresponding author upon reasonable request.

## Supplementary Materials

The following are available online at https://www.mdpi.com/article/10.3390/life11070641/s1: Table S1, Primers used to amplify the main globozoospermia-associated genes; Table S2, Sperm parameters of globozoospermic patients; Table S3, Mean ± SD, median (in brackets) and significance of the sperm parameters between complete and partial form of globozoospermia (Mann–Whitney U test). Significant *p* values are in bold; Table S4, Mean ± SD, median (in brackets) and significance of the sperm parameters between globozoospermic samples of men with and without *DPY19L2* deletions (Mann–Whitney U test). Significant *p* values are in bold.

## References

[1] Pallotti F., Paoli D., Carlini T., Vestri A.R., Martino G., Lenzi A., Lombardo F. (2018). Varicocele and semen quality: A retrospective case-control study of 4230 patients from a single centre. J. Endocrinol. Investig..

[2] Corona G., Sansone A., Pallotti F., Ferlin A., Pivonello R., Isidori A.M., Maggi M., Jannini E.A. (2020). People smoke for nicotine, but lose sexual and reproductive health for tar: A narrative review on the effect of cigarette smoking on male sexuality and reproduction. J. Endocrinol. Investig..

[3] Holstein A.F., Schirren C., Schirren C.G. (1973). Human spermatids and spermatozoa lacking acrosomes. J. Reprod. Fertil..

[4] Dam A.H.D.M., Feenstra I., Westphal J.R., Ramos L., van Golde R.J., Kremer J.A. (2007). Globozoospermia revisited. Hum. Reprod. Update.

[5] Rubino P., Viganò P., Luddi A., Piomboni P. (2016). The ICSI procedure from past to future: A systematic review of the more controversial aspects. Hum. Reprod. Update.

[6] Chansel-Debordeaux L., Dandieu S., Bechoua S., Jimenez C. (2015). Reproductive outcome in globozoospermic men: Update and prospects. Andrology.

[7] Schirren C.G., Holstein A.F., Schirren C. (1971). Über die Morphogenese rund-köpfiger Spermatozoen des Menschen. Andrologia.

[8] Singh G. (1992). Ultrastructural features of round-headed human spermatozoa. Int. J. Fertil..

[9] Dam A.H.D.M., Ramos L., Dijkman H.B., Woestenenk R., Robben H., van den Hoven L., Kremer J.A. (2011). Morphology of partial globozoospermia. J. Androl..

[10] Blanchard Y., Lescoat D., Le Lannou D. (1990). Anomalous distribution of nuclear basic proteins in round-headed human spermatozoa. Andrologia.

[11] Yassine S., Escoffier J., Martinez G., Coutton C., Karaouzène T., Zouari R., Ravanat J.-L., Metzler-Guillemain C., Lee H.C., Fissore R. (2015). Dpy19 l2-deficient globozoospermic sperm display altered genome packaging and DNA damage that compromises the initiation of embryo development. Mol. Hum. Reprod..

[12] Vicari E., Perdichizzi A., De Palma A., Burrello N., D’Agata R., Calogero A.E. (2002). Globozoospermia is associated with chromatin structure abnormalities: Case report. Hum. Reprod..

[13] Gatimel N., Leandri R.D., Foliguet B., Bujan L., Parinaud J. (2013). Sperm cephalic vacuoles: New arguments for their non acrosomal origin in two cases of total globozoospermia. Andrology.

[14] Vozdova M., Rybar R., Kloudova S., Prinosilova P., Texl P., Rubes J. (2014). Total globozoospermia associated with increased frequency of immature spermatozoa with chromatin defects and aneuploidy: A case report. Andrologia.

[15] Perrin A., Coat C., Nguyen M.H., Talagas M., Morel F., Amice J., De Braekeleer M. (2013). Molecular cytogenetic and genetic aspects of globozoospermia: A review. Andrologia.

[16] De Braekeleer M., Nguyen M.H., Morel F., Perrin A. (2015). Genetic aspects of monomorphic teratozoospermia: A review. J. Assist. Reprod. Genet..

[17] Liu G., Shi Q.-W., Lu G.-X. (2010). A newly discovered mutation in PICK1 in a human with globozoospermia. Asian J. Androl..

[18] Dam A.H.D.M., Koscinski I., Kremer J.A.M., Moutou C., Jaeger A.-S., Oudakker A.R., Tournaye H., Charlet N., Lagier-Tourenne C., van Bokhoven H. (2007). Homozygous mutation in SPATA16 is associated with male infertility in human globozoospermia. Am. J. Hum. Genet..

[19] Harbuz R., Zouari R., Pierre V., Ben Khelifa M., Kharouf M., Coutton C., Merdassi G., Abada F., Escoffier J., Nikas Y. (2011). A recurrent deletion of DPY19L2 causes infertility in man by blocking sperm head elongation and acrosome formation. Am. J. Hum. Genet..

[20] Koscinski I., Elinati E., Fossard C., Redin C., Muller J., Velez de la Calle J., Schmitt F., Ben Khelifa M., Ray P.F., Ray P. (2011). DPY19L2 deletion as a major cause of globozoospermia. Am. J. Hum. Genet..

[21] Coutton C., Zouari R., Abada F., Ben Khelifa M., Merdassi G., Triki C., Escalier D., Hesters L., Mitchell V., Levy R. (2012). MLPA and sequence analysis of DPY19L2 reveals point mutations causing globozoospermia. Hum. Reprod..

[22] Pierre V., Martinez G., Coutton C., Delaroche J., Yassine S., Novella C., Pernet-Gallay K., Hennebicq S., Ray P.F., Arnoult C. (2012). Absence of Dpy19l2, a new inner nuclear membrane protein, causes globozoospermia in mice by preventing the anchoring of the acrosome to the nucleus. Development.

[23] Tang T., Li L., Tang J., Li Y., Lin W.Y., Martin F., Grant D., Solloway M., Parker L., Ye W. (2010). A mouse knockout library for secreted and transmembrane proteins. Nat. Biotechnol..

[24] Chianese C., Fino M.G., Riera Escamilla A., Lòpez Rodrigo O., Vinci S., Guarducci E., Daguin F., Muratori M., Tamburrino L., Lo Giacco D. (2015). Comprehensive investigation in patients affected by sperm macrocephaly and globozoospermia. Andrology.

[25] Coutton C., Escoffier J., Martinez G., Arnoult C., Ray P.F. (2015). Teratozoospermia: Spotlight on the main genetic actors in the human. Hum. Reprod. Update.

[26] WHO (2010). WHO Laboratory Manual for the Examination and Processing of Human Semen.

[27] Carlini T., Paoli D., Pelloni M., Faja F., Dal Lago A., Lombardo F., Lenzi A., Gandini L. (2017). Sperm DNA fragmentation in Italian couples with recurrent pregnancy loss. Reprod. Biomed. Online.

[28] Paoli D., Pecora G., Pallotti F., Faja F., Pelloni M., Lenzi A., Lombardo F. (2019). Cytological and molecular aspects of the ageing sperm. Hum. Reprod..

[29] Kang-Decker N., Mantchev G.T., Juneja S.C., McNiven M.A., van Deursen J.M. (2001). Lack of acrosome formation in Hrb-deficient mice. Science.

[30] Yao R., Ito C., Natsume Y., Sugitani Y., Yamanaka H., Kuretake S., Yanagida K., Sato A., Toshimori K., Noda T. (2002). Lack of acrosome formation in mice lacking a Golgi protein, GOPC. Proc. Natl. Acad. Sci. USA.

[31] Xiao N., Kam C., Shen C., Jin W., Wang J., Lee K.M., Jiang L., Xia J. (2009). PICK1 deficiency causes male infertility in mice by disrupting acrosome formation. J. Clin. Investig..

[32] Fesahat F., Henkel R., Agarwal A. (2020). Globozoospermia syndrome: An update. Andrologia.

[33] Larson K.L., Brannian J.D., Singh N.P., Burbach J.A., Jost L.K., Hansen K.P., Kreger D.O., Evenson D.P. (2001). Chromatin structure in globozoospermia: A case report. J. Androl..

[34] Ghasemzadeh J., Talebi A.R., Khalili M.A., Fesahat F., Halvaei I., Nabi A., Ashourzadeh S. (2015). Sperm parameters, protamine deficiency, and apoptosis in total globozoospermia. Iran. J. Reprod. Med..

[35] Hosseinifar H., Yazdanikhah S., Modarresi T., Totonchi M., Sadighi Gilani M., Sabbaghian M. (2015). Correlation between sperm DNA fragmentation index and CMA 3 positive spermatozoa in globozoospermic patients. Andrology.

[36] Eskandari N., Tavalaee M., Zohrabi D., Nasr-Esfahani M.H. (2018). Association between total globozoospermia and sperm chromatin defects. Andrologia.

[37] Talebi A.R., Ghasemzadeh J., Khalili M.A., Halvaei I., Fesahat F. (2018). Sperm chromatin quality and DNA integrity in partial versus total globozoospermia. Andrologia.

[38] Tavalaee M., Nomikos M., Lai F.A., Nasr-Esfahani M.H. (2018). Expression of sperm PLCζ and clinical outcomes of ICSI-AOA in men affected by globozoospermia due to DPY19L2 deletion. Reprod. Biomed. Online.

[39] Florke-Gerloff S., Topfer-Petersen E., Muller-Esterl W., Mansouri A., Schatz R., Schirren C., Schill W., Engelal W. (1984). Biochemical and genetic investigation of round-headed spermatozoa in infertile men including two brothers and their father. Andrologia.

[40] Carrell D.T., Emery B.R., Liu L. (1999). Characterization of aneuploidy rates, protamine levels, ultrastructure, and functional ability of roundheaded sperm from two siblings and implications for intracytoplasmic sperm injection. Fertil. Steril..

[41] Carrell D.T., Wilcox A.L., Udoff L.C., Thorp C., Campbell B. (2001). Chromosome 15 aneuploidy in the sperm and conceptus of a sibling with variable familial expression of round-headed sperm syndrome. Fertil. Steril..

[42] Kilani Z., Ismail R., Ghunaim S., Mohamed H., Hughes D., Brewis I., Barratt C.L.R. (2004). Evaluation and treatment of familial globozoospermia in five brothers. Fertil. Steril..

[43] Dirican E.K., Isik A., Vicdan K., Sozen E., Suludere Z. (2008). Clinical pregnancies and livebirths achieved by intracytoplasmic injection of round headed acrosomeless spermatozoa with and without oocyte activation in familial globozoospermia: Case report. Asian J. Androl..

[44] Lu L., Lin M., Xu M., Zhou Z.M., Sha J.H. (2006). Gene functional research using polyethylenimine-mediated in vivo gene transfection into mouse spermatogenic cells. Asian J. Androl..

[45] Fujihara Y., Oji A., Larasati T., Kojima-Kita K., Ikawa M. (2017). Human Globozoospermia-Related Gene Spata16 Is Required for Sperm Formation Revealed by CRISPR/Cas9-Mediated Mouse Models. Int. J. Mol. Sci..

[46] Ellnati E., Fossard C., Okutman O., Ghédir H., Ibala-Romdhane S., Ray P.F., Saad A., Hennebicq S., Viville S. (2016). A new mutation identified in SPATA16 in two globozoospermic patients. J. Assist. Reprod. Genet..

[47] Ghédir H., Braham A., Viville S., Saad A., Ibala-Romdhane S. (2019). Comparison of sperm morphology and nuclear sperm quality in SPATA16- and DPY19L2-mutated globozoospermic patients. Andrologia.

[48] Modarres P., Tanhaei S., Tavalaee M., Ghaedi K., Deemeh M.R., Nasr-Esfahani M.H. (2016). Assessment of DPY19L2 Deletion in Familial and Non-Familial Individuals with Globozoospermia and DPY19L2 Genotyping. Int. J. Fertil. Steril..

[49] Ellnati E., Kuentz P., Redin C., Jaber S., Vanden Meerschaut F., Makarian J., Koscinski I., Nasr-Esfahani M.H., Demirol A., Gurgan T. (2012). Globozoospermia is mainly due to DPY19L2 deletion via non-allelic homologous recombination involving two recombination hotspots. Hum. Mol. Genet..

[50] Noveski P., Madjunkova S., Maleva I., Sotiroska V., Petanovski Z., Plaseska-Karanfilska D. (2013). A Homozygous Deletion of the DPY19l2 Gene is a Cause of Globozoospermia in Men from the Republic of Macedonia. Balkan J. Med. Genet..

[51] Zhu F., Gong F., Lin G., Lu G. (2013). DPY19L2 gene mutations are a major cause of globozoospermia: Identification of three novel point mutations. Mol. Hum. Reprod..

[52] Ounis L., Zoghmar A., Coutton C., Rouabah L., Hachemi M., Martinez D., Martinez G., Bellil I., Khelifi D., Arnoult C. (2015). Mutations of the aurora kinase C gene causing macrozoospermia are the most frequent genetic cause of male infertility in Algerian men. Asian J. Androl..

[53] Ghédir H., Ibala-Romdhane S., Okutman O., Viot G., Saad A., Viville S. (2016). Identification of a new DPY19L2 mutation and a better definition of DPY19L2 deletion breakpoints leading to globozoospermia. Mol. Hum. Reprod..

[54] Shang Y.L., Zhu F.X., Yan J., Chen L., Tang W.H., Xiao S., Mo W.K., Zhang Z.G., He X.J., Qiao J. (2019). Novel DPY19L2 variants in globozoospermic patients and the overcoming this male infertility. Asian J. Androl..

[55] Baccetti B., Collodel G., Piomboni P. (1996). Apoptosis in human ejaculated sperm cells. J. Submicrosc. Cytol. Pathol..

[56] Tejera A., Molla M., Muriel L., Remohi J., Pellicer A., De Pablo J.L. (2008). Successful pregnancy and childbirth after intracytoplasmic sperm injection with calcium ionophore oocyte activation in a globozoospermic patient. Fertil. Steril..

[57] Egashira A., Murakami M., Haigo K., Horiuchi T., Kuramoto T. (2009). A successful pregnancy and live birth after intracytoplasmic sperm injection with globozoospermic sperm and electrical oocyte activation. Fertil. Steril..

[58] Taylor S.L., Yoon S.Y., Morshedi M.S., Lacey D.R., Jellerette T., Fissore R.A., Oehninger S. (2010). Complete globozoospermia associated with PLCzeta deficiency treated with calcium ionophore and ICSI results in pregnancy. Reprod. Biomed. Online.

[59] Brahem S., Mehdi M., Elghezal H., Saad A. (2011). Analysis of sperm aneuploidies and DNA fragmentation in patients with globozoospermia or with abnormal acrosomes. Urology.

[60] Perrin A., Louanjli N., Ziane Z., Louanjli T., Le Roy C., Guéganic N., Amice V., De Braekeleer M., Morel F. (2011). Study of aneuploidy and DNA fragmentation in gametes of patients with severe teratozoospermia. Reprod. Biomed. Online.

[61] Zhioua A., Merdassi G., Bhouri R., Ferfouri F., Ben Ammar A., Amouri A., Vialard F., Zhioua F. (2011). Apport de l’exploration cytogénétique et ultrastructurale dans le pronostic de fertilité des sujets globozoospermiques. Andrologie.

[62] Haghighat S., Tavalaee M., Kouhkan A., Zakeri Z., Noureddini M., Shahverdi A.H., Nasr Esfahani M.H. (2019). Reduction of truncated Kit Expression in men with abnormal semen parameters, globozoospermia and history of low or fertilization failure. Cell, J..

[63] Moretti E., Collodel G., Salvatici M.C., Belmonte G., Signorini C. (2019). New insights into sperm with total globozoospermia: Increased fatty acid oxidation and centrin1 alteration. Syst. Biol. Reprod. Med..

[64] Sermondade N., Hafhouf E., Dupont C., Bechoua S., Palacios C., Eustache F., Poncelet C., Benzacken B., Lévy R., Sifer C. (2011). Successful childbirth after intracytoplasmic morphologically selected sperm injection without assisted oocyte activation in a patient with globozoospermia. Hum. Reprod..

[65] Grassetti D., Paoli D., Gallo M., D’Ambrosio A., Lombardo F., Lenzi A., Gandini L. (2012). Protamine-1 and -2 polymorphisms and gene expression in male infertility: An Italian study. J. Endocrinol. Investig..

[66] Lundin K., Sjögren A., Nilsson L., Hamberger L. (1994). Fertilization and pregnancy after intracytoplasmic microinjection of acrosomeless spermatozoa. Fertil. Steril..

[67] Stone S., O’Mahony F., Khalaf Y., Taylor A., Braude P. (2000). A normal live birth after intracytoplasmic sperm injection for globozoospermia without assisted oocytes activation: Case report. Hum. Reprod..

[68] Zeyneloglu H.B., Baltaci V., Duran H.E., Erdemli E., Batioglu S. (2002). Achievement of pregnancy in globozoospermia with Y chromosome microdeletion after ICSI. Hum. Reprod..

[69] Battaglia D.E., Koehler J.K., Klein N.A., Tucker M.J. (1997). Failure of oocyte activation after intracytoplasmic sperm injection using round-headed sperm. Fertil. Steril..

[70] Sfontouris I.A., Nastri C.O., Lima M.L., Tahmasbpourmarzouni E., Raine-Fenning N., Martins W.P. (2015). Artificial oocyte activation to improve reproductive outcomes in women with previous fertilization failure: A systematic review and meta-analysis of RCTs. Hum. Reprod..

[71] Fawzy M., Emad M., Mahran A., Sabry M., Fetih A.N., Abdelghafar H., Rasheed S. (2018). Artificial oocyte activation with SrCl2 or calcimycin after ICSI improves clinical and embryological outcomes compared with ICSI alone: Results of a randomized clinical trial. Hum. Reprod..

[72] Murugesu S., Saso S., Jones B.P., Bracewell-Milnes T., Athanasiou T., Mania A., Serhal P., Ben-Nagi J. (2017). Does the use of calcium ionophore during artificial oocyte activation demonstrate an effect on pregnancy rate? A meta-analysis. Fertil. Steril..

[73] Dam A.H., Pijnenburg A.J., Hendriks J.C., Westphal H., Ramos L., Kremer J.A. (2012). Intracytoplasmic sperm injection in partial globozoospermia. Fertil. Steril..

[74] Kuentz P., Vanden Meerschaut F., Elinati E., Nasr-Esfahani M.H., Gurgan T., Iqbal N., Carré-Pigeon F., Brugnon F., Gitlin S.A., Velez de la Calle J. (2013). Assisted oocyte activation overcomes fertilization failure in globozoospermic patients regardless of the DPY19L2 status. Hum. Reprod..

[75] Karaca N., Yilmaz R., Kanten G.E., Kervancioglu E., Solakoglu S., Kervancioglu M.E. (2014). First successful pregnancy in a globozoospermic patient having homozygous mutation in SPATA16. Fertil. Steril..

[76] Canepa P., Casciano I., De Leo C., Massarotti C., Anserini P., Remorgida V., Scaruffi P. (2019). A successful healthy childbirth and an ongoing evolutive pregnancy in a case of partial globozoospermia by hyaluronic acid sperm selection. Andrologia.

[77] Kochhar P.K., Ghosh P. (2018). Intracytoplasmic Sperm Injection with Assisted Oocyte Activation Resulting in Successful Pregnancies and Live Birth in Couples with Globozoospermia: A Report of Two Cases. J. Hum. Reprod. Sci..

[78] Niu X., Ruan Q., Witz C.A., Wang W. (2020). Comparison of Human Oocyte Activation Between Round-Headed Sperm Injection Followed by Calcium Ionophore Treatment and Normal Sperm Injection in a Patient With Globozoospermia. Front. Endocrinol..

[79] Ghazavi F., Peymani M., Hashemi M.S., Ghaedi K., Nasr-Esfahani M.H. (2019). Embryos derived from couples with consanguineous marriages with globozoospermia should be screened for gender or DPY19L2 deletion. Andrologia.

