# Supplementary material for: Molecular Analysis of DPY19L2, PICK1 and SPATA16 in Italian Unrelated Globozoospermic Men
Source: Life (Basel). 2021 Jun 30;11(7):641. doi: 10.3390/life11070641 (PMC8307282; doi:10.3390/life11070641)
Supplement: Supplementary file 1 [file life-11-00641-s001.zip › life-1244259-supplementary.pdf]

life

Article

Molecular Analysis of *DPY19L2*, *PICK1* and *SPATA16* in Italian Unrelated Globozoospermic Men

Fabiana Faja, Francesco Pallotti, Francesco Cargnelutti, Giulia Senofonte, Tania Carlini, Andrea Lenzi, Francesco Lombardo and Donatella Paoli *

Laboratory of Seminology—"Loredana Gandini" Sperm Bank, Department of Experimental Medicine, "Sapienza" University of Rome, Viale del Policlinico 155, 00161 Rome, Italy; fabianafaja@hotmail.it (F.F.); francesco.pallotti@uniroma1.it (F.P.); francesco.cargnelutti@uniroma1.it (F.C.); giulia.senofonte@uniroma1.it (G.S.); tania.carlini@uniroma1.it (T.C.); andrea.lenzi@uniroma1.it (A.L.); francesco.lombardo@uniroma1.it (F.L.)

* Correspondence: donatella.paoli@uniroma1.it; Tel.: +3390649970715

Abstract: This study aims to evaluate genetic contribution and sperm DNA fragmentation (SDF) in a cohort of 18 unrelated globozoospermic Italian men (Group G). Semen samples were assessed according to the WHO 2010 Laboratory Manual and compared with 31 fertile controls. We focused our genetic analysis on the exons of the main globozoospermia-associated genes, performing qualitative PCR to assess deletion of *DPY19L2* and sequencing to detect mutations of *SPATA16* and *PICK1*. SDF was evaluated using the TUNEL assay. In Group G, 10 patients had a complete form of globozoospermia, whereas 8 patients had a partial form. Molecular analysis revealed deletion of *DPY19L2* in six of the patients, all of them with complete globozoospermia, while no mutations were found in the examined exons of *PICK1* and *SPATA16*. TUNEL analysis showed a higher SDF% in Group G. Our findings confirm *DPY19L2* defects as the most frequent genetic alteration in Italian patients contributing to globozoospermic phenotypes. Furthermore, spermatozoa with acrosomal defects could also display high levels of SDF as a possible consequence of abnormally remodeled chromatin. The possible effect on offspring of chromatin structure abnormalities and altered DNA integrity should be carefully evaluated by clinicians, especially regarding the feasibility and safety of artificial reproductive techniques, which represent the only treatment that allows these patients to conceive.

Keywords: globozoospermia; acrosome; male infertility; genetics; sequencing; sperm DNA fragmentation

Citation: Faja, F.; Pallotti, F.; Cargnelutti, F.; Senofonte, G.; Carlini, T.; Lenzi, A.; Lombardo, F.; Paoli, D. Molecular Analysis of *DPY19L2*, *PICK1* and *SPATA16* in Italian Unrelated Globozoospermic Men. *Life* **2021**, *11*, 641. https://doi.org/10.3390/life11070641

Academic Editors: Renata Walczak-Jedrzejowska, Małgorzata Piasecka and Jolanta Słowikowska-Hilczer

Received: 17 May 2021
Accepted: 25 June 2021
Published: 30 June 2021

Publisher's Note: MDPI stays neutral with regard to jurisdictional claims in published maps and institutional affiliations.

Copyright: © 2021 by the authors. Licensee MDPI, Basel, Switzerland. This article is an open access article distributed under the terms and conditions of the Creative Commons Attribution (CC BY) license (http://creativecommons.org/licenses/by/4.0/).

Supplementary Materials:

Table S1. Primers used to amplify the main globozoospermia-associated genes.

Gene	Exons Examined	Primers	Reference
SPATA16	exon 4	F 5′-CCTGTGATTTCATTCCATT-3′<br>R 5′-TGTTATGCTATTACCAGAA-3′	[53]
PICK1	exon 13	F 5′-CTCCTGCGTTCCCTGAACTG-3′<br>R 5′-CCTCGTGTATCCCTGGACGG-3′	[17]
DPY19L2	exon 1	F 5′-GGCCAACTTCTTTCTACTCGGAC-3′<br>R 5′-ATTTCACAGTCGCCATGACG-3′	[21]
	exon 10	F 5′-CCAAAGAGGAGGTACCGTATAA-3′<br>R 5′-GCCATCCATCTTTTTAATTCTG-3′	[21]
	exon 11	F 5′-AACCTCCTCAAGTGACTTAG-3′<br>R 5′-TTGGCCAAGAGTCATT-3′	[21]
	exon 12	F 5′-GAAGGTTAATTGAAGCTAGA-3′<br>R 5′-ATTAGCCTGCAGAAAATGGT-3′	[21]
	exon 20	F 5′-CAGAGGCAACAGGTACGTAT-3′<br>R 5′-ACCCTTAGAACTGTGAAGATTA-3′	[21]
	exon 22	F 5′-CTTTATTATTAGGATATGTCTTTCCC-3′<br>R 5′-TTACCTTTTAGTAATCAGAAAAATTTC-3′	[49]

Table S2. Sperm parameters of globozoospermic patients.

	Semen volume (ml)	Sperm Concentration (10^6/ml)	Total Sperm Number (10^6/ejaculate)	Progressive Motility (%)	Abnormal Forms (%)
**Globo 1**	1.8	55.0	99.0	55	90
**Globo 2**	3.5	28.0	98.0	35	100
**Globo 3**	0.9	25.0	22.5	15	100
**Globo 4**	3.0	58.0	174.0	40	100
**Globo 5**	2.8	90.0	252.0	40	100
**Globo 6**	2.5	60.0	150.0	50	80
**Globo 7**	0.5	140.0	70.0	40	100
**Globo 8**	3.2	66.0	211.2	40	100
**Globo 9**	5.0	18.0	90.0	30	90
**Globo 10**	1.5	40.0	60.0	45	90
**Globo 11**	2.2	120.0	264.0	45	100
**Globo 12**	1.0	310.0	310.0	35	100
**Globo 13**	1.2	20.0	24.0	15	95
**Globo 14**	3.0	75.0	225.0	35	100
**Globo 15**	4.2	12.0	50.4	20	100
**Globo 16**	4.5	120.0	540.0	40	80
**Globo 17**	2.5	80.0	200.0	35	90
**Globo 18**	2.5	35.0	87.5	40	88

Table S3. Mean ± SD, median (in brackets) and significance of the sperm parameters between complete and partial form of globozoospermia (Mann–Whitney U test). Significant *P* values are in bold.

Form of Globozoospermia	Semen Volume (ml)	Sperm Concentration (10^6/ml)	Total Sperm Number (10^6/ejaculate)	Progressive Motility (%)	Abnormal Forms (%)
**Complete**	2.4 ± 1.2 (2.9)	92.4 ± 86.7 (70.5)	167.7 ± 100.6 (192.6)	34.5 ± 9.6 (37.5)	100.0 ± 0.0 (100.0)
**Partial**	2.7 ± 1.4 (2.5)	53.5 ± 34.0 (47.5)	156.3 ± 164.0 (94.5)	38.8 ± 12.5 (40.0)	87.9 ± 5.2 (90.0)
***P* value**	0.965	0.315	0.515	0.408	**< 0.001**

Table S4. Mean ± SD, median (in brackets) and significance of the sperm parameters between globozoospermic samples of men with and without *DPY19L2* deletions (Mann–Whitney U test). Significant *P* values are in bold.

DPY19L2 Deletions	Semen Volume (ml)	Sperm Concentration (10^6/ml)	Total Sperm Number (10^6/ejaculate)	Progressive Motility (%)	Abnormal Forms (%)
**Present**	2.4 ± 1.5 (2.6)	66.7 ± 53.9 (51.5)	121.7 ± 99.1 (84.0)	31.7 ± 11.7 (35.0)	100.0 ± 0.0 (100.0)
**Absent**	2.6 ± 1.2 (2.5)	79.3 ± 78.3 (59.0)	183.1 ± 140.1 (162.0)	38.8 ± 10.0 (40.0)	91.9 ± 7.3 (90.0)
***P* value**	0.750	0.820	0.385	0.250	**0.024**
